# Functional Near-Infrared Spectroscopy Reveals Functional Rewiring between Macaque Motor Areas Following Postinfarction Recovery of Manual Dexterity

**DOI:** 10.1523/JNEUROSCI.1400-25.2025

**Published:** 2025-12-04

**Authors:** Jun-ichiro Hirayama, Toru Yamada, Hiroshi Kawaguchi, Noriyuki Higo, Narihisa Matsumoto

**Affiliations:** ^1^National Institute of Advanced Industrial Science and Technology (AIST), Tsukuba, Ibaraki 305-8568, Japan; ^2^University of Tsukuba, Tsukuba, Ibaraki 305-8577, Japan

**Keywords:** fNIRS, functional connectivity, Granger causality, macaque monkey, poststroke, rehabilitation

## Abstract

Poststroke motor recovery relies on the functional reorganization of motor-related cortical areas to compensate for damaged neural circuitry related to motor functions. Functional near-infrared spectroscopy (fNIRS) offers a promising method for monitoring cortical reorganization during rehabilitation, although its feasibility has not yet been fully established. We developed a high-density fNIRS system for measuring cortical activity in macaques and validated its effectiveness in assessing changes in directed functional connectivity (dFC) among motor cortical areas in response to functional recovery from brain damage. Data were previously collected from two female Japanese macaque monkeys (*Macaca fuscata*) before focal infarcts occurred in the posterior limb of the internal capsule and following the confirmed recovery of hand movements through rehabilitation training. In the present study, time-varying conditional Granger causality during either unaffected- or affected-hand movement was evaluated among several motor areas, and the changes resulting from functional impairment and subsequent recovery were analyzed. Changes in channel-level dFC around the ventral premotor areas were examined in detail for their relevance to motor recovery. The analysis revealed network changes involving multiple motor-related cortical regions. In the monkey with a small infarct, connectivity changes primarily occurred within the ipsilesional hemisphere, whereas in the monkey with a larger infarct, both contralesional and interhemispheric connectivity change was observed. These results align with the findings obtained from high-spatial-resolution brain measurements in patients and animal models following brain damage. They suggest that functional connectivity analysis using fNIRS is effective for monitoring the brain’s functional changes underlying motor recovery.

## Significance Statement

We analyzed brain activity in macaque monkeys both before the induction of focal infarcts in the internal capsule and after the recovery of hand movements, using functional near-infrared spectroscopy (fNIRS). Directed functional connectivity (dFC) among motor-related cortical areas during hand movement tasks was assessed using time-varying conditional Granger causality. The results revealed task-dependent dFC among several motor areas, indicating network changes (functional rewiring) involving multiple regions. Notably, a small infarct primarily affected connectivity within the ipsilesional hemisphere, whereas a larger infarct involved both contralesional and interhemispheric connections. The results of the present study suggest that task-dependent functional connectivity changes measured by fNIRS can be used to assess cortical reorganization associated with poststroke motor recovery.

## Introduction

Motor recovery from brain damage depends on the brain's ability to functionally reorganize to compensate for the loss of functionality in damaged neural tissues. Neuroimaging studies of patients with stroke and other types of brain damage, along with physiological and anatomical studies of animal models with artificially induced brain damage, have revealed that the activity of brain areas spared from damage increases to compensate for the functions of the affected regions (Johansen-Berg et al., 2002; Hsu and Jones, 2006; Liu et al., 2008; Schaechter and Perdue, 2008; Bestmann et al., 2010; Rehme et al., 2011; Bradnam et al., 2012; Kantak et al., 2012; Nudo, 2013; Higo, 2014; Touvykine et al., 2016; Dodd et al., 2017; Jones, 2017; Higo, 2021; Yu et al., 2024). The structure and function of the macaque motor cortex and motor output pathways are more similar to those of humans than other experimental animals (Courtine et al., 2007; Lemon, 2008), and macaque monkeys possess the largest brain among all experimental animals, allowing brain imaging data acquisition comparable to that in humans. Therefore, research using macaque monkey models of brain damage is highly significant for translational research in patients with brain damage. Our previous study, using positron emission tomography scans in macaque monkeys, reported increased activation of the ventral premotor cortex (PMv) after the recovery of dexterous hand movements following ibotenic acid lesioning of the primary motor cortex (M1; Murata et al., 2015). Our study using functional near-infrared spectroscopy (fNIRS) also reported increased activation of the PMv after hand motor recovery from focal infarcts in the posterior limb of the internal capsule (Kato et al., 2020).

In addition to increased brain activation, previous studies have reported that changes in functional connectivity between brain areas also contribute to the recovery of motor function after brain damage (Grefkes and Fink, 2014; Cassidy et al., 2022; Ismail et al., 2024). Resting-state functional magnetic resonance imaging (rsfMRI) has been used to evaluate functional connectivity during rest (rs-FC) in patients with brain damage. The advantage of rsfMRI is that it can be used with patients who have severe motor paralysis because it can be performed without motor tasks. In the present study, we focused on task-dependent directed functional connectivity (dFC), measured by time-varying conditional Granger causality (tvGC) during a small-object retrieval task. This analysis allowed us to observe dynamic interactions between functional modules related to a specific task, thus offering greater sensitivity in detecting changes in neural circuits involved in the recovery of specific functions compared with rs-FC. There are fewer reports on task-dependent functional connectivity changes during motor recovery than on rs-FC (Grefkes and Fink, 2014; Cassidy et al., 2022; Ismail et al., 2024). Furthermore, we focused on fNIRS, which is less expensive and less physically constraining than fMRI, to explore its application in physical motor rehabilitation. Another advantage of fNIRS over fMRI is its higher temporal resolution, allowing for more detailed Granger causality (GC) analysis. Therefore, this study demonstrates the feasibility of using fNIRS to monitor cortical reorganization associated with motor recovery after brain damage.

Experimental studies using brain-damaged animal models offer the advantage of comparing functional connectivity following functional recovery with measurements taken before brain damage in the same subjects. Moreover, animal models allow for the creation of focal damage, making it possible to clarify the relationship between damaged regions and changes in functional connectivity that underlie the recovery of specific functions. We investigated the feasibility of evaluating task-dependent functional connectivity changes after brain damage using fNIRS. We used a macaque model of focal infarcts in the posterior limb of the internal capsule (Murata and Higo, 2016) and a high-density fNIRS measurement system that we previously established for monitoring macaque cortical activity (Murata and Higo, 2016; Yamada et al., 2018).

## Materials and Methods

### Experimental design

We reanalyzed the fNIRS data originally acquired in a previous study (Kato et al., 2020). The data were recorded from two Japanese macaque monkeys (Monkey 1 and Monkey 2), while they performed a manual dexterity task. While seated in a primate chair, the monkey retrieved a small spherical object (food pellet) with either the left or right hand from a cylindrical well in a Klüver board (Klüver, 1935). Each monkey normally performed 150 task trials (75 for each hand) during a daily session. The size of the well varied across sessions (fixed within each session), either 10, 11, or 20 mm in diameter (5 mm in depth), although we did not use the size information in the analysis reported here.

Each monkey completed 12 daily sessions of fNIRS measurement before focal infarcts were induced by injecting endothelin-1 into the posterior limb of the internal capsule in the left hemisphere (Murata and Higo, 2016), which impaired movement of the right hand. Therefore, we refer to the left and right hemispheres as the ipsilesional and contralesional hemispheres, respectively, and the left and right hands, including those prior to infarction, as the unaffected and affected hands, respectively. Recovery of dexterous affected-hand movements was then confirmed after more than a month of rehabilitation training, and a further 12 sessions of fNIRS measurement were conducted. Therefore, there were four experimental conditions, prelesion-unaffected, prelesion-affected, postrecovery-unaffected, and postrecovery-affected, referring to unaffected-hand and affected-hand use preinfarction and postrecovery.

The experiment was approved by the Institutional Animal Care and Use Committee of the National Institute of Advanced Industrial Science and Technology, and it was implemented under the “Guide for the Care and Use of Laboratory Animals” (Eighth ed., National Research Council of the National Academies).

### Data acquisition

**Figure 1.**
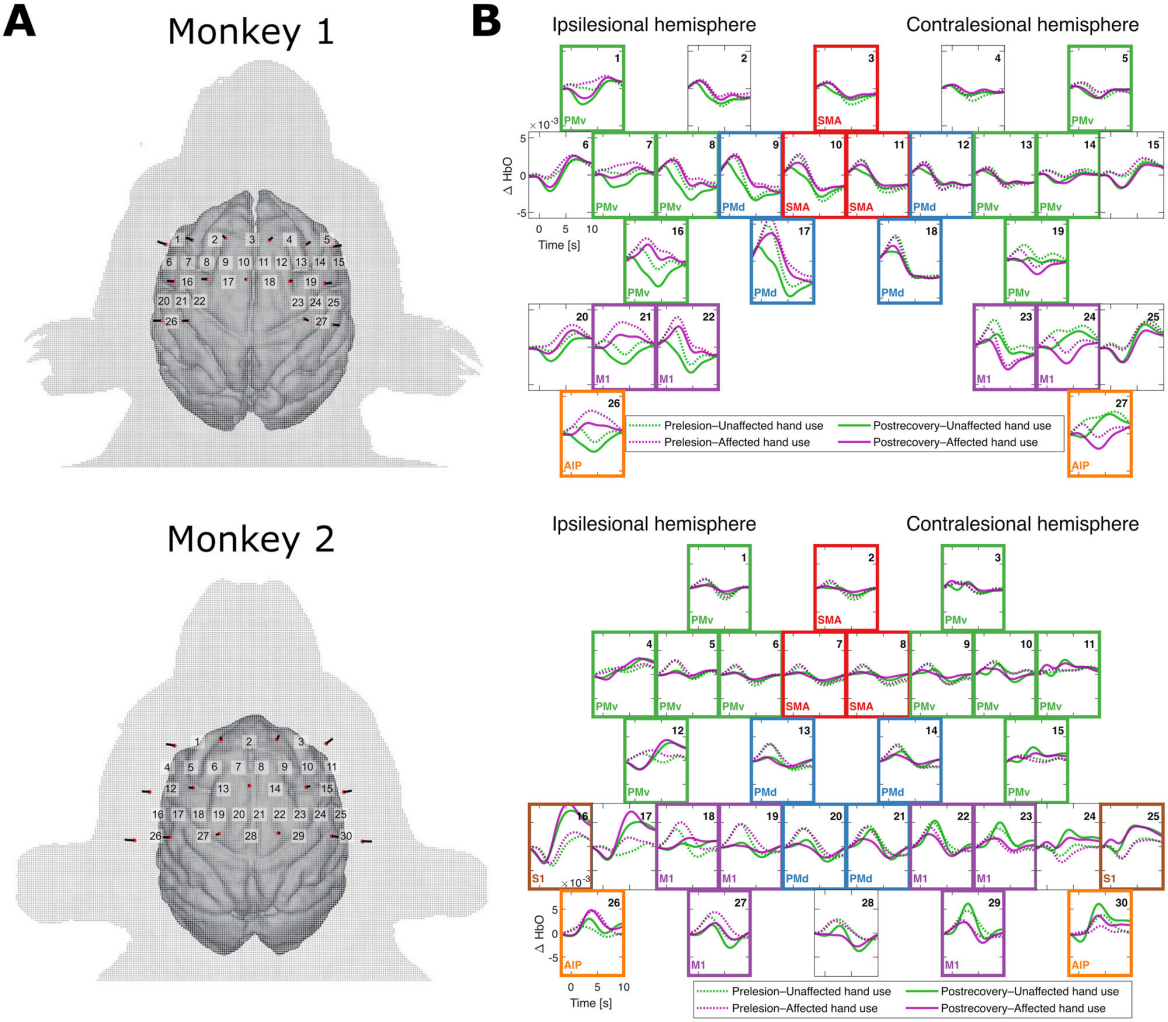
***A***, Spatial layouts of fNIRS channels (indicated by the numbers), each located between a pair of optodes, adapted from Kato et al. (2020; CC BY 4.0). ***B***, Trial-averaged ΔHbO signals in each of the four experimental conditions and the correspondence between the fNIRS channels and anatomical ROIs. The number on the top right of each panel indicates the corresponding channel in ***A***. The text in the bottom left and the frame color of a panel indicate the corresponding ROI. Top, Monkey 1. Bottom, Monkey 2. SMA, supplementary motor area; PMd, dorsal premotor cortex; PMv, ventral premotor cortex; M1, primary motor area; AIP, anterior intraparietal area; S1, primary somatosensory cortex.

A high-density fNIRS measurement system (Yamada et al., 2018; Kato et al., 2020) was used to monitor cerebral functional hemodynamic responses. In both monkeys, the scalp was incised under pentobarbital anesthesia, and optode sockets were formed on the skull surface using self-curing acrylic resin mixed with titanium oxide to match the optical scattering coefficient of the skull. The spatial arrangement of the optodes (Fig. 1*A*) was determined based on the anatomical information from T1- and T2-weighted MR images, ensuring that the optodes covered the majority of the motor-related cortical areas. A triangular bidirectional measurement approach (Yamada et al., 2018; Kato et al., 2020) was used to detect fNIRS signals at each channel, incorporating pairs of adjacent optodes, with a high spatial density of several millimeters and a sampling interval of 0.13 s. See Kato et al. (2020) for more details regarding the fNIRS measurements, as well as information on the subjects and experimental protocols.

### fNIRS signal preprocessing

In the present study, we focused on changes in oxygenated hemoglobin (ΔHbO) as a measure of brain activity. Each ΔHbO signal was first bandpass filtered (0.02–0.3 Hz, fourth-order zero–phase Butterworth filter), and a constant was subtracted to set the initial value to zero. For each measurement channel, the two ΔHbO signals corresponding to the bidirectional optical pathways between the paired optodes were averaged. The resultant (averaged) ΔHbO signals for the 27 and 30 channels for Monkey 1 and Monkey 2, respectively, were further epoched by extracting the [−5, 15] s period around each trial onset and then baseline-corrected by subtracting the mean of the [−5, 0] s period from each trial. The trial onsets were set to the time when the monkey’s upper limb movements were detected by a digital laser sensor at the slit in front of the food well. The total number of trials was 620, 619, 824, and 824 for Monkey 1 and 898, 898, 857, and 857 for Monkey 2 across the prelesion-unaffected, prelesion-affected, postrecovery-unaffected, and postrecovery-affected conditions, respectively. The trial-averaged ΔHbO signals for each condition are shown in Figure 1*B*, where the spatial layout corresponds to the relative channel locations between a pair of optodes for each monkey (Kato et al., 2020).

### Anatomical regions of interest

Several regions of interest (ROIs) were defined based on anatomical considerations for each monkey. The correspondence between the channels and ROIs is depicted in Figure 1*B*. The common ROIs for both monkeys were the supplementary motor area (SMA), ipsilesional/contralesional dorsal premotor cortices (ip-PMd/co-PMd), ipsilesional/contralesional ventral premotor cortices (ip-PMv/co-PMv), ipsilesional/contralesional primary motor areas (ip-M1/co-M1), and ipsilesional/contralesional anterior intraparietal areas (ip-AIP/co-AIP). Owing to differences in the coverage of the optode arrays, the ipsilesional/contralesional primary somatosensory cortices (ip-S1/co-S1) were specific to Monkey 2. Some channels could not be clearly identified as belonging to any ROI or were found to measure mixed signals from more than one ROI. These channels were excluded from the definitions of ROIs; however, they were used to compute the channel-level pairwise conditional GC, as introduced in the next section, to attenuate their confounding effects. The subsequent region-level analyses described below were conducted primarily using these ROIs, although some results were also reproduced with modified ROIs in which the caudal channels in PMv and PMd (i.e., Channels 16, 17, 18, and 19 in Monkey 1 and 12, 15, 20, and 21 in Monkey 2) were removed, as depicted in Figure S1.

### Channel-level dFC

Channel-level dFC was measured using the tvGC for each monkey across each of the four experimental conditions. First, we fitted a time-varying multivariate autoregressive (tvMAR) model to all instances (trials) of the multichannel ΔHbO signals for the [−5, 10] s period relative to the trial onset. The tvMAR model is defined as follows:

x(t)=A(1,t)x(t−1)+A(2,t)x(t−2)+⋯+A(p,t)x(t−p)+ϵ(t),

where x(t) denotes the signal vector containing the channel-wise ΔHbO signals at discrete time point *t*, A(k,t) represents the time-dependent AR coefficient matrices at the time point lags of k=1,2,…,p, and ϵ(t) is a zero-mean white noise vector with covariance $\Sigma_{\epsilon }$. The AR order *p* was fixed at 15 (1.95 s) in this study. The coefficient matrices are assumed to vary smoothly over time, modeled by a random walk process:

A(k,t)=A(k,t−1)+ω(t),

where ω(t) is a zero-mean white noise matrix with covariance $\Sigma_{\omega }$. To estimate the time-varying coefficient matrices A(k,t) and noise covariance $\Sigma_{\epsilon }$, we used a MATLAB implementation of the tvMAR model fitting (https://github.com/PscDavid/dynet_toolbox) called the self-tuning optimized Kalman filter (Pascucci et al., 2020).

Then, conditional GC was evaluated for each ordered pair of channels at each time point *t* based on the obtained tvMAR model. The pairwise GC index from source channel *i* to target channel *j* is defined at a specific time point *t* as follows:

$$GC_{i\to j}(t):=\ln\frac{\sigma_{j}^{\setminus i}(t)}{\sigma_{j}(t)},$$

where $\sigma_{j}(t)$ and $\sigma_{j}^{\setminus i}(t)$ denote the residual error variances of the target channel (signal) *j* given all the other channels and all the other channels excluding the source channel *i*, respectively. We used the MVGC toolbox (Barnett and Seth, 2014) in MATLAB to compute the latter efficiently using a frequency-domain technique without re-estimating a reduced tvMAR model that excludes the effects of the source channel *i*. Ultimately, we identified time-varying directed functional networks for each of the prelesion-unaffected, prelesion-affected, postrecovery-unaffected, and postrecovery-affected conditions, where every directed connectivity $GC_{i\to j}(t)$ is non-negative because $\sigma_{j}^{\setminus i}(t)$ cannot be smaller than $\sigma_{j}(t)$ by definition. The number of directed connections, i.e., ordered pairs (*i*, *j*), for i≠j, was 702 for Monkey 1 and 870 for Monkey 2.

### Region-level summaries of pre–post dFC changes

The pre–post changes in the channel-level dFC were summarized at the ROI level as follows. Following the reorganization of the neural network after stroke, both increases and decreases in dFC may occur between specific regions. When a region consists of multiple channels, channel-level dFC can be used to distinguish between increases and decreases in region-level dFC. Therefore, we decided to summarize the increases and decreases separately. Specifically, we first computed the pre–post differences in directed connectivity $GC_{i\to j}(t)$—that is, channel-level GC changes—for every *i* in a particular region (denoted as A) and *j* in another region (denoted as B) in either the unaffected- or affected-hand use condition. Then, we evaluated the largest positive and smallest negative differences—region-level GC increases and decreases, respectively—to summarize the channel-level changes in GC from Region A to Region B in a time-resolved manner. Alternatively, we computed the region-level increases and decreases in GC in a time-integrated (or time-windowed) manner by first summing only the positive and negative channel-level changes in GC over time and then evaluating their largest positive and smallest negative values, respectively. We used either the entire time window of [−2, 10] s to characterize the total changes or the half-overlapping 4 s time windows centered at 0, 2, 4, and 8 s, i.e., [−2, 2], [0, 4], [2, 6], [4, 8], and [6, 10] s, to reveal time-dependent differences in the pre–post connectivity change. The region-level increases or decreases in GC, denoted generically by $\Delta GC^{+}\;(\geq 0)$ or $\Delta GC^{-}\;(\leq 0)$, were set to zero if all corresponding channel-level changes in GC or their summations over time were nonpositive or non-negative, respectively.

To summarize, we quantified the pre–post changes in dFC between regions, either in a time-resolved manner or appropriately time-integrated (time-windowed), based on the four measures, ${\Delta \mathrm{GC}}_{\mathrm{Unaffected}}^{+}$, ${\Delta \mathrm{GC}}_{\mathrm{Unaffected}}^{-}$, ${\Delta \mathrm{GC}}_{\mathrm{Affected}}^{+}$, and ${\Delta \mathrm{GC}}_{\mathrm{Affected}}^{-}$, i.e., the region-level increases and decreases in GC for the unaffected-hand and affected-hand use conditions. To highlight the total relative changes associated with affected-hand use, we also evaluated the relative increases ${\Delta \mathrm{GC}}_{\mathrm{Affected}}^{+}-{\Delta \mathrm{GC}}_{\mathrm{Unaffected}}^{+}$ and decreases $\left|{\Delta \mathrm{GC}}_{\mathrm{Affected}}^{-}|-|{\Delta \mathrm{GC}}_{\mathrm{Unaffected}}^{-}\right|$ in GC, time-integrated over the entire window (i.e., [−2, 10] s). Both types of changes were defined as positive if they were more pronounced for affected-hand than for unaffected-hand use. To facilitate interpretation, we depicted the time-integrated (time-windowed) region-level increases and decreases in GC, as well as the relative changes, as edge weights in directed graphs with each node representing a single ROI. Additionally, we evaluated the mean connectivity changes within and between hemispheres in each monkey by averaging the time-integrated (total) increases and decreases in GC, i.e., ${\Delta \mathrm{GC}}_{\mathrm{Unaffected}}^{+}$, ${\Delta \mathrm{GC}}_{\mathrm{Unaffected}}^{-}$, ${\Delta \mathrm{GC}}_{\mathrm{Affected}}^{+}$, and ${\Delta \mathrm{GC}}_{\mathrm{Affected}}^{-}$, over all statistically significant connections (see below) between every corresponding pair of distinct ROIs (in both directions), with the SMA regarded as being in both hemispheres.

### Channel-level dFC changes around PMv cortices

Kato et al. (2020) reported that pharmacological inactivation of local areas around Channels 8 and 16 (ip-PMv, Monkey 1) or Channel 11 (co-PMv, Monkey 2) again impaired the affected-hand movements in both monkeys, suggesting their causal relevance to motor recovery. For direct comparison with their original finding, we further examined the channel-level GC changes related to those “seed” channels. In addition, we evaluated the total channel-level increases in GC—the sum of only positive channel-level GC changes over the entire time window of [−2, 10] s—for each unaffected- and affected-hand use condition across all ordered pairs of channels within any ROI. We focused on increases because they primarily reflect functional reorganization associated with restoring motor output pathways.

For the channel-by-channel matrix of statistically significant (see below, Statistical analysis) total channel-level increases in GC, which we treated as an adjacency matrix of a weighted graph, we ranked the importance of the channels, especially the seed channels, according to their degree centralities. These were calculated as the sum of incoming (in-degree) and outgoing (out-degree) edge weights in the directed graph for each channel. Finally, for improved intuitive understanding, we summarized the pre–post increases in GC at the region level (integrated within the time window of [−2, 10] s) and depicted them as directed graphs. Here, each target channel—Channels 1, 7, 8, and 16 (ip-PMv) in Monkey 1 and Channels 3, 9, 10, 11, and 15 (co-PMv) in Monkey 2 (Fig. 1)—was treated as a single node for a closer view of the relevant PMv area. The computational procedure was the same as above, except that the target channels in ip-PMv/co-PMv were treated separately.

### Statistical analysis

To test the statistical significance of the region- and channel-level changes (or relative changes) in GC, whether measured through the time-resolved or the time-integrated approach, we performed permutation tests as follows. First, we created 1,000 surrogate datasets by randomly permuting the prelesion/postrecovery labels for all trials corresponding to each unaffected- and affected-hand use condition. Next, we fitted the tvMAR model and evaluated the channel-level changes in GC for each surrogate dataset, resulting in 1,000 instances of the changes of interest in GC for every relevant pair of ROIs or channels. Finally, their *p* values were computed as the number of surrogate instances that were greater than or equal to the original values in absolute terms, divided by 1,000. Statistical significance was assessed collectively for all scalar quantities of interest in each analysis (e.g., across all ROI/channel pairs and/or time points/windows), using a false discovery rate (FDR) threshold of 0.1 (Benjamini and Hochberg, 1995).

## Results

### Pre–post changes in dFC among motor-related cortices

**Figure 2.**
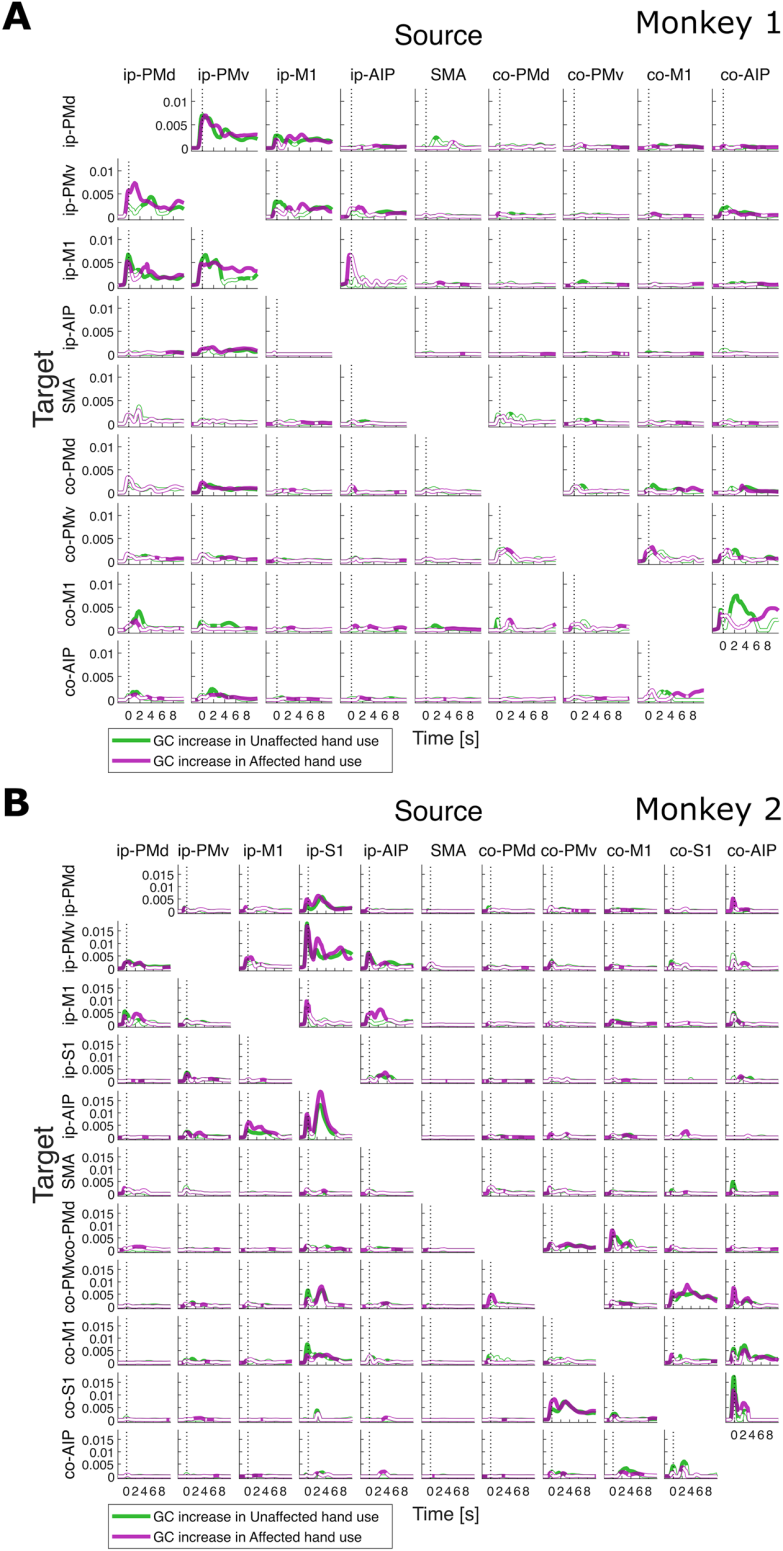
Pre–post increase in region-level time–varying GC for (***A***) Monkey 1 and (***B***) Monkey 2. Green and purple curves correspond to unaffected-hand and affected-hand uses, respectively. Curves filled with the respective colors indicate statistical significance and those filled with white indicate nonsignificance (permutation test, FDR = 0.1). Each small panel shows the pre–post increases or decreases for a particular pair of source and target ROIs, as indicated, evaluated at every time point between [−2, 10] s, where 0 s is the detected movement onset (vertical dotted line).

**Figure 3.**
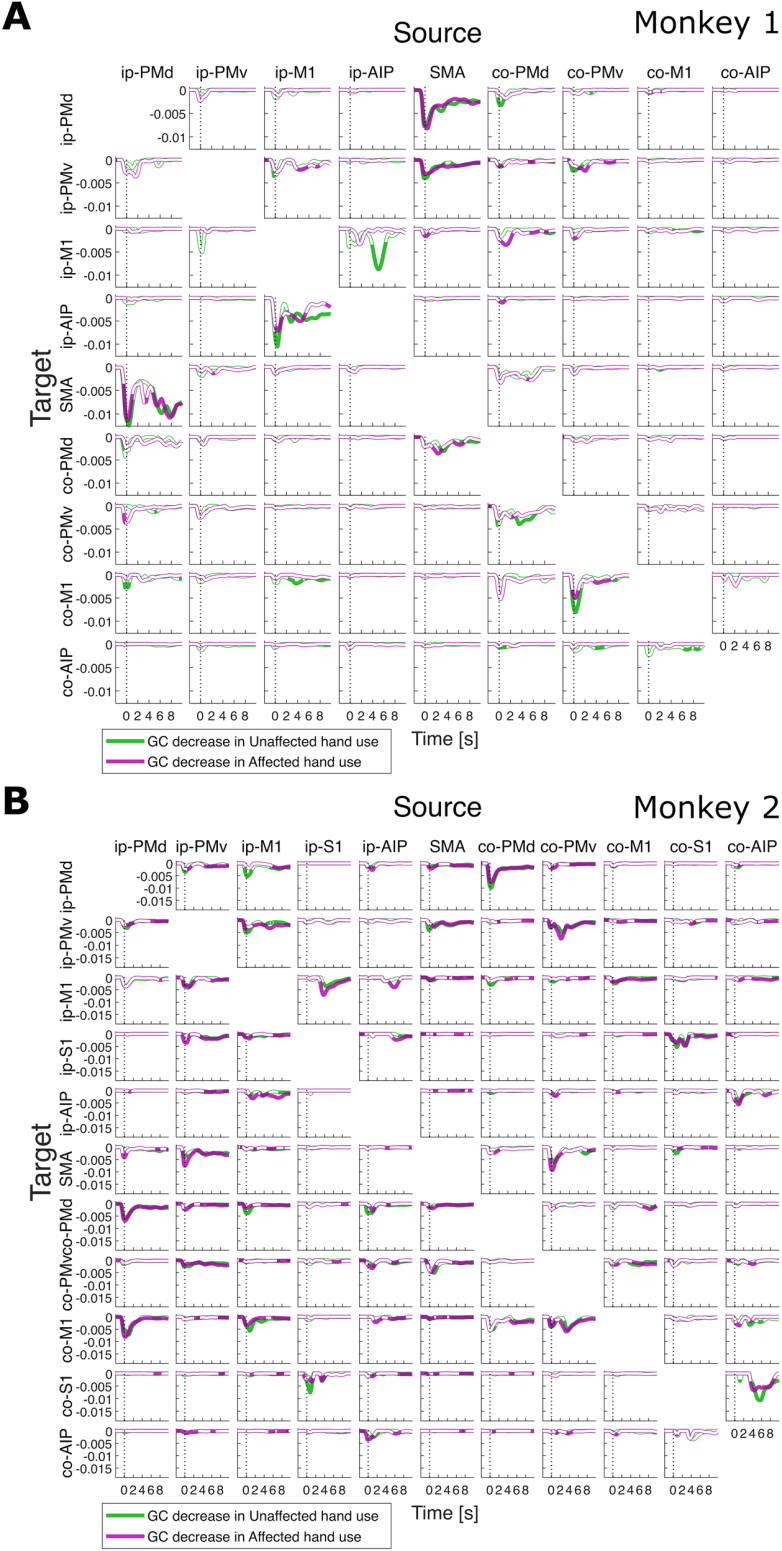
Pre–post decrease in region-level time–varying GC for (***A***) Monkey 1 and (***B***) Monkey 2. Green and purple curves correspond to unaffected and affected hand use, respectively. Curves filled with the respective colors indicate statistical significance and those filled with white indicate nonsignificance (permutation test, FDR = 0.1). Each small panel shows the pre–post increases or decreases for a particular pair of source and target ROIs, as indicated, evaluated at every time point between [−2,10] s, where 0 s is the detected movement onset (vertical dotted line).

First, we examined the changes in dFC among motor cortical regions, measured as the greatest increases and decreases in channel-level tvGC between two regions (i.e., region-level GC changes), from the preinfarction state in the left internal capsule to the postfunctional recovery phase of dexterous affected (right)-hand movements in each monkey. The region-level changes in GC for both the unaffected- (left) and affected-hand uses are summarized as time-resolved waveforms in Figure 2 (increases) and Figure 3 (decreases). In Monkey 1, large increases in GC were found in the connections between the ip-PMv and its neighboring regions, ip-PMd and ip-M1, shortly after trial onset (Fig. 2*A*). The changes in GC were relatively small between the hemispheres and within the contralesional hemisphere, except for a notable increase in the connection from co-AIP to co-M1. In contrast, connections with surrounding areas in the same hemisphere, such as those between ip-PMd and SMA and between ip-M1 and ip-AIP, showed a decrease (Fig. 3*A*). In Monkey 2, increases in GC were evident after trial onset in both the ipsilesional and contralesional hemispheres, corresponding to the top-left and bottom-right blocks (separated by the SMA) of the panels in Figure 2*B*, respectively. The increases in GC were pronounced for some connections, such as between PMv and S1 in both hemispheres and from ip-S1 to ip-AIP. Conversely, the decreases were characterized by relatively large interhemispheric connections, corresponding to the diagonal panels within each off-diagonal block (Fig. 3*B*).

**Figure 4.**
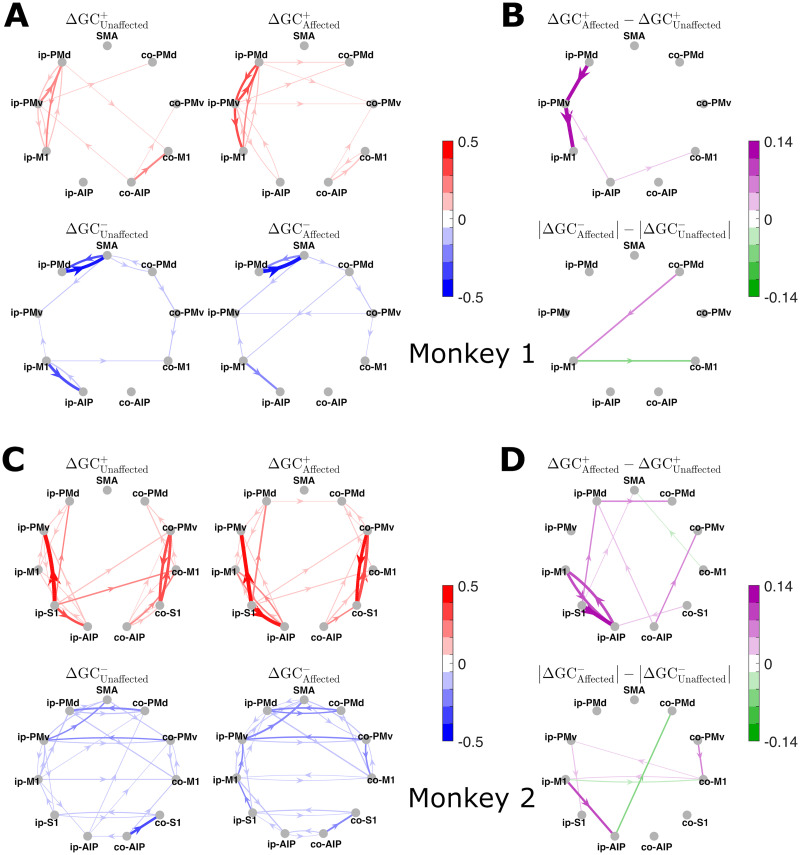
***A***, Directed graphs displaying the total increases (top row) and decreases (bottom row) in GC within [−2, 10] s for unaffected-hand (unaffected) and affected-hand (affected) use conditions in Monkey 1 and (***B***) the differences (i.e., relative increases and decreases in GC) between unaffected and affected. Only statistically significant edges are shown (permutation test, FDR = 0.1). For clarity, significant but small connectivity values are not shaded (color levels in white). ***C, D***, The same as for Monkey 2.

The time-integrated (total) increases and decreases in GC are depicted as directed graphs in Figure 4*A* (Monkey 1) and Figure 4*C* (Monkey 2), summarizing the entire [−2, 10] s interval, with task onset designated as the temporal origin. In Monkey 1, these directed graphs confirmed that the large increases and decreases were primarily focused on the connections around PMv and neighboring areas within the ipsilesional hemisphere. Notably, the directed graphs for unaffected-hand and affected-hand use are quite similar, although they exhibit characteristic differences as indicated by the additional directed graphs representing the relative changes in GC (Fig. 4*B*). These graphs show that the increases in GC between ip-PMv and its neighboring areas were more prominent for affected-hand than unaffected-hand use, whereas the decreases in GC in the connections between ip-PMd/ip-M1 and surrounding areas were not significantly different between unaffected- and affected-hand use. However, the differences in decreases in GC between unaffected-hand and affected-hand use were significant for interhemispheric connections, such as those from co-PMd to ip-M1 (greater decrease for affected-hand use) and from ip-M1 to co-M1 (greater decrease for unaffected-hand use). In Monkey 2, the total changes in GC summarized by the directed graphs (Fig. 4*C*) also confirmed the observations above. Again, the directed graphs were quite similar for unaffected-hand and affected-hand use, whereas the graphs showing the relative changes in GC (Fig. 4*D*) clearly indicate that the increases, such as from ip-M1 to ip-AIP and ip-S1 to ip-AIP, were more prominent for affected-hand than unaffected-hand use. It should be noted that the characteristic decreases in GC in the interhemispheric connections were not significantly different between unaffected-hand and affected-hand use. Relatively large decreases in GC for affected-hand use were suggested, such as for the connection from ip-M1 to ip-AIP.

**Figure 5.**
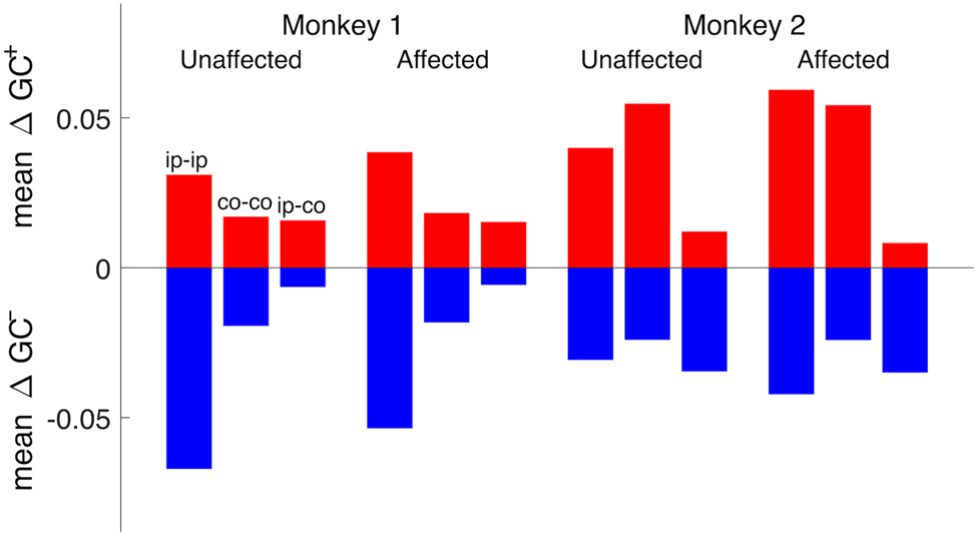
Mean connectivity changes within and between hemispheres corresponding to the eight directed graphs in Figure 4, *A* and *C*, for the unaffected-hand (unaffected) and affected-hand (affected) use conditions in both monkeys. In each of the four corresponding blocks, the three pairs of red and blue bars represent the mean total changes in GC for the connections within the ipsilesional hemisphere (ip-ip), within the contralesional hemisphere (co-co), and between the hemispheres (ip-co), ordered from the left to the right; red and blue indicate increases and decreases, respectively. Nonsignificant connectivity changes were regarded as zero when computing the means. Both the increases and decreases exhibit similar patterns between unaffected and affected in both monkeys but are rather different between monkeys.

The directed graphs of total GC changes, shown in Figure 4*A* and Figure 4*C*, are further summarized with their mean changes in GC within and between hemispheres in Figure 5. This figure confirms that the overall patterns of total GC changes were quite similar between the unaffected-hand and affected-hand use conditions in both monkeys, whereas they were rather different between monkeys. Indeed, as noted earlier, both increases and decreases were focused within the ipsilesional hemisphere in Monkey 1, whereas in Monkey 2 they were observed in both the ipsilesional and contralesional hemispheres. Additionally, decreases in GC between the two hemispheres were especially prominent in Monkey 2.

**Figure 6.**
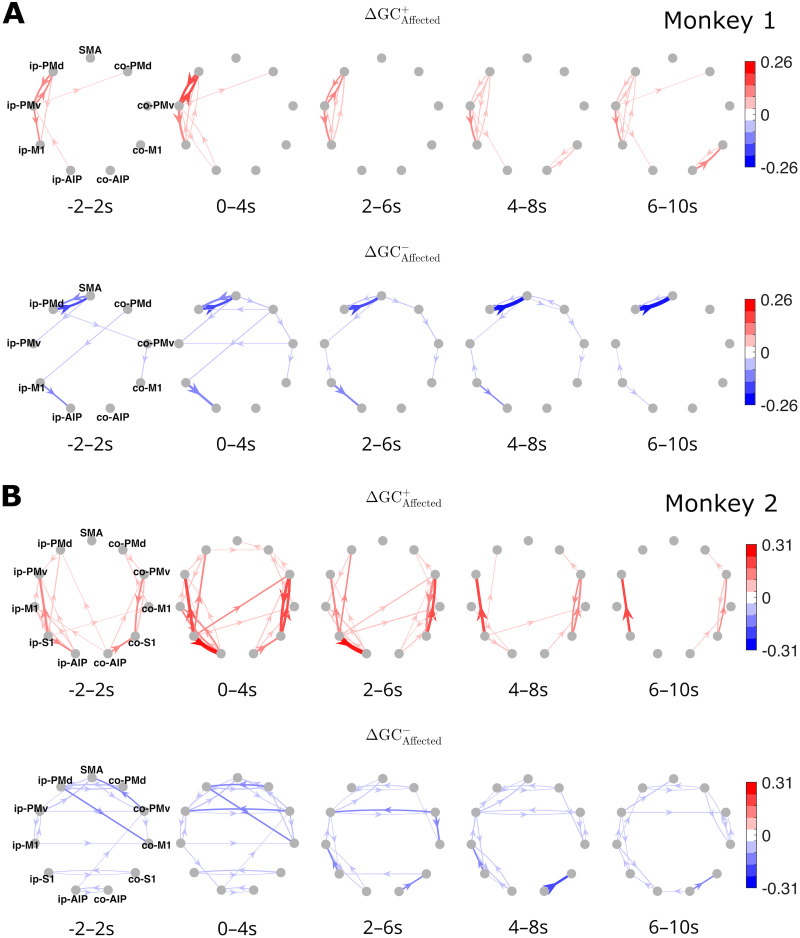
Directed graphs displaying time-windowed region–level increases and decreases in GC for affected-hand use in (***A***) Monkey 1 and (***B***) Monkey 2. The increases and decreases were time-integrated for each 4 s half-overlapping time window, as indicated under each graph. Directed edges in red and blue represent positive and negative edge weights, respectively, with darker colors indicating stronger weights. Only statistically significant edges are shown in each graph (permutation test, FDR = 0.1). The common color limit ranges from the negative to the positive of the absolute maximum weights across the 10 graphs in this figure. For clarity, significant but small connectivity values are not shaded (color levels in white).

Additional time-windowed directed graphs in Figure 6*A* (Monkey 1) and Figure 6*B* (Monkey 2), focusing on affected-hand use, provide a more intuitive illustration of the time-dependent differences in region-level pre–post changes in GC than their corresponding time-resolved waveforms. In Monkey 1, the directed graphs suggest that the increases in GC within the ipsilesional hemisphere, particularly between ip-PMd and ip-PMv, were concentrated in the early stages (e.g., 0–4 s). In Monkey 2, they indicate, for example, that the increase from co-PMv to co-S1 started earlier (i.e., it was greater at −2 to 2 s) than the reverse connection from co-S1 to co-PMv. Similarly, the decrease in GC from co-PMv to ip-PMv started earlier than the reverse connection from ip-PMv to co-PMv.

To gain insights into how region-level changes in GC rely on the selection of ROIs, we reproduced their time-resolved waveforms (Figs. S2 and S3) and time-integrated directed graphs (Fig. S4), using the modified ROIs in which caudal channels in PMv/PMd (i.e., those close to M1/S1) were intentionally removed (Fig. S1). The results of this reanalysis were largely consistent with the original findings, encompassing most of the features described earlier. The only notable difference was a reduction in the increase in GC between ip-PMv/ip-PMd and ip-M1 in Monkey 1 (Fig. S4). Considering previous evidence that the caudal regions of PMv/PMd project anatomically to the hand area of M1, this difference seems reasonable (Dum and Strick, 2005). While the current analysis demonstrates a degree of robustness regarding ROI selection, it also highlights the need for investigations of GC changes at the level of individual subregions.

### Channel-level dFC changes around PMv areas causally relevant to motor recovery

In the original Kato et al. (2020) study, pharmacological inactivation of local areas around Channels 8 and 16 (ip-PMv, Monkey 1) or Channel 11 (co-PMv, Monkey 2) again impaired the affected-hand movements in both monkeys. To connect their findings with our dFC analysis, we next examined the channel-level changes in GC around these local PMv areas in more detail.

**Figure 7.**
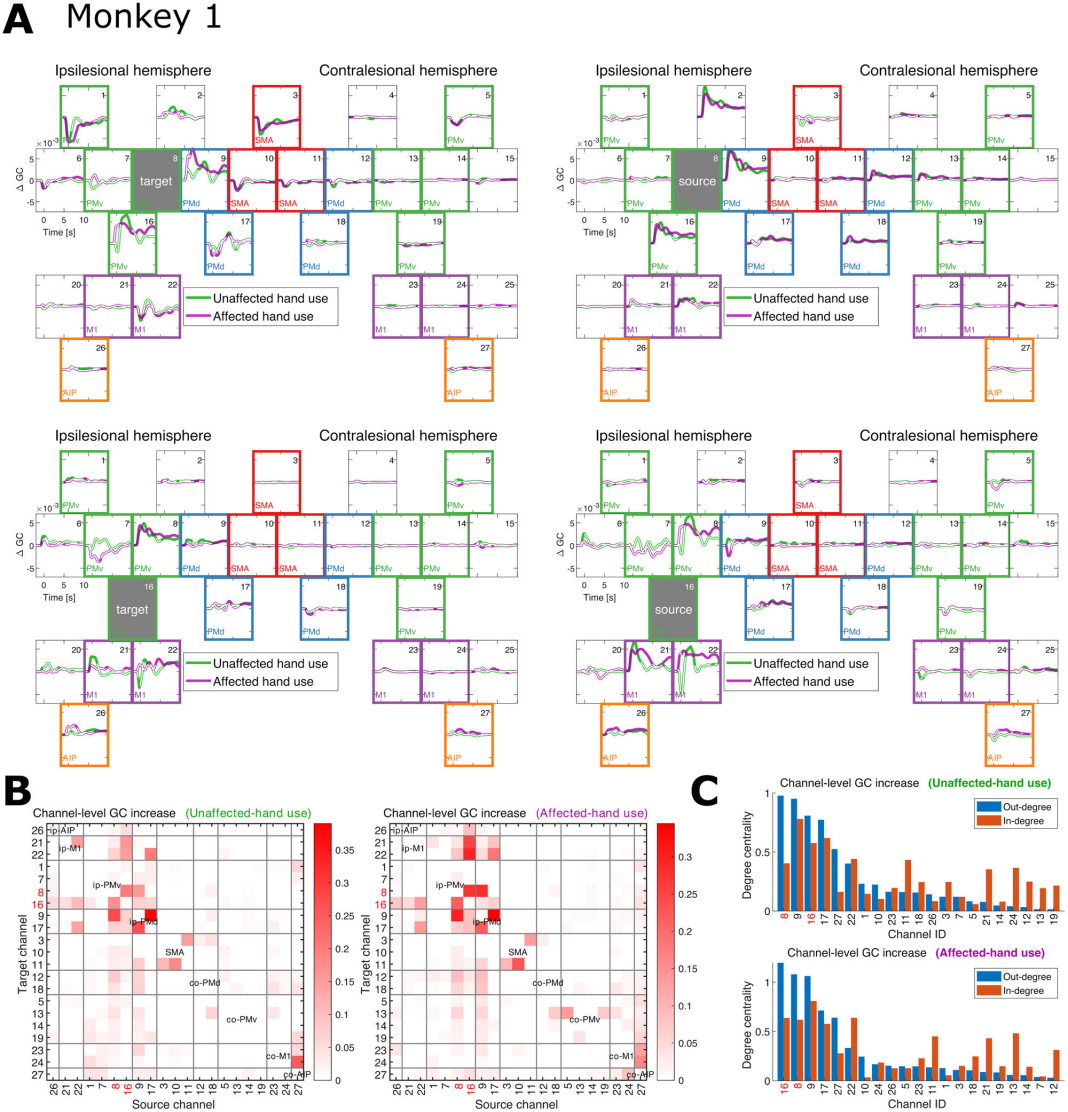
Monkey 1. ***A***, Time-resolved channel-level pre–post changes in GC for Channels 8 and 16, representing the source or target of directed connectivity. These channels (local areas in ip-PMv) were selected because their causal relevance to motor recovery was experimentally confirmed by Kato et al. (2020). ***B***, Time-integrated (total) channel-level GC increases for each pair of channels located within any ROI in the unaffected-hand and affected-hand use conditions. In both conditions, Channels 8 and 16 (indicated by numbers in red) exhibit relatively large increases in GC, especially within the off-diagonal (between-ROI) blocks of the ipsilesional hemisphere. ***C***, The corresponding degree centralities (i.e., sums of incoming or outgoing edge weights) when viewing each matrix in (***B***) as an adjacency (weight) matrix of a directed graph. The large out-degrees of Channels 8 and 16 (numbers in red) indicate their potential causal relevance.

For Monkey 1, the time-resolved channel–level GC changes related to Channels 8 and 16 (ip-PMv) are shown in Figure 7*A*. The increases in unaffected-hand or affected-hand use (or both) are particularly evident in the connections from Channels 9 (ip-PMd) and 16 (ip-PMv) to Channel 8 and from Channel 8 to Channels 9 (ip-PMd) and 16 (ip-PMv), as well as those from Channel 8 to Channel 16 and from Channel 16 to Channels 8, 21 (ip-M1), and 22 (ip-M1), among others. Integrated over time, these connections exhibited particularly large (total) channel-level GC increases compared with the others, as represented by the channel-by-channel weight (adjacency) matrices (Fig. 7*B*). Channels 8 and 16 were further characterized by their relatively large out-degree centralities (i.e., column sums of the weight matrices of the directed graph) in the corresponding directed graphs (Fig. 7*C*). This suggests that these channels played a major role in the pre–post dFC changes, with their causal effects on other local areas increasing along with functional recovery.

**Figure 8.**
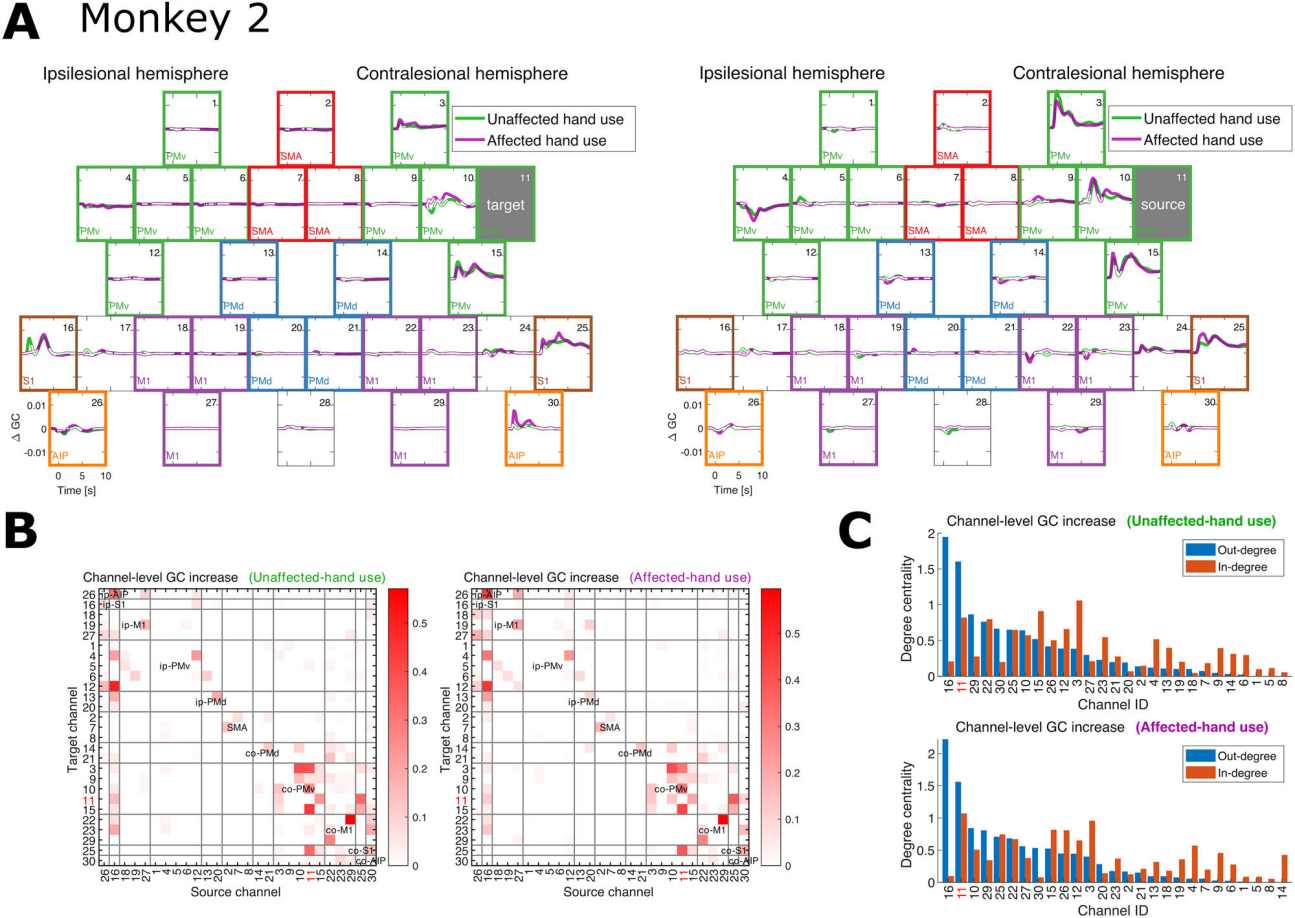
Monkey 2. ***A***, Time-resolved channel–level pre–post GC changes for Channel 11 representing the source or target of directed connectivity. This channel (local area in co-PMv) was selected because its causal relevance to motor recovery was experimentally confirmed by Kato et al. (2020). ***B***, Time-integrated (total) channel-level GC increases for every pair of channels located within any ROI in unaffected- and affected-hand use conditions. In both conditions, Channel 11 (indicated by numbers in red) exhibits relatively large increases in GC, especially within the off-diagonal (between-ROI) blocks of the contralesional hemisphere. ***C***, The corresponding degree centralities (i.e., sums of incoming or outgoing edge weights) when viewing each matrix in ***B*** as an adjacency (weight) matrix of a directed graph. The large out-degrees of Channel 11 (numbers in red) and Channel 16 indicate their potential causal relevance.

For Monkey 2, the time-resolved channel–level changes in GC related to Channel 11 (co-PMv) are shown in Figure 8*A*. The increases are evident in the connections from Channels 3, 10, 15 (co-PMv), 16 (ip-S1), 25 (co-S1), and 30 (co-AIP) to Channel 11 and from Channel 11 to Channels 3, 10, 15 (co-PMv), and 25 (co-S1). The time-integrated (total) channel-level GC increases, represented by the channel-by-channel weight (adjacency) matrices (Fig. 8*B*), indicate relatively large increases within-co-PMv connections, including those related to Channel 11 and the connection between Channel 11 and Channel 25 (co-S1), as well as some connections from Channel 16 (ip-S1) within the ipsilesional hemisphere. Indeed, Channels 16 and 11 exhibited the first and second largest out-degrees in the corresponding directed graphs (Fig. 8*C*). Again, this suggests that Channel 11 is one of the key channels representing the changes in dFC related to the functional recovery.

**Figure 9.**
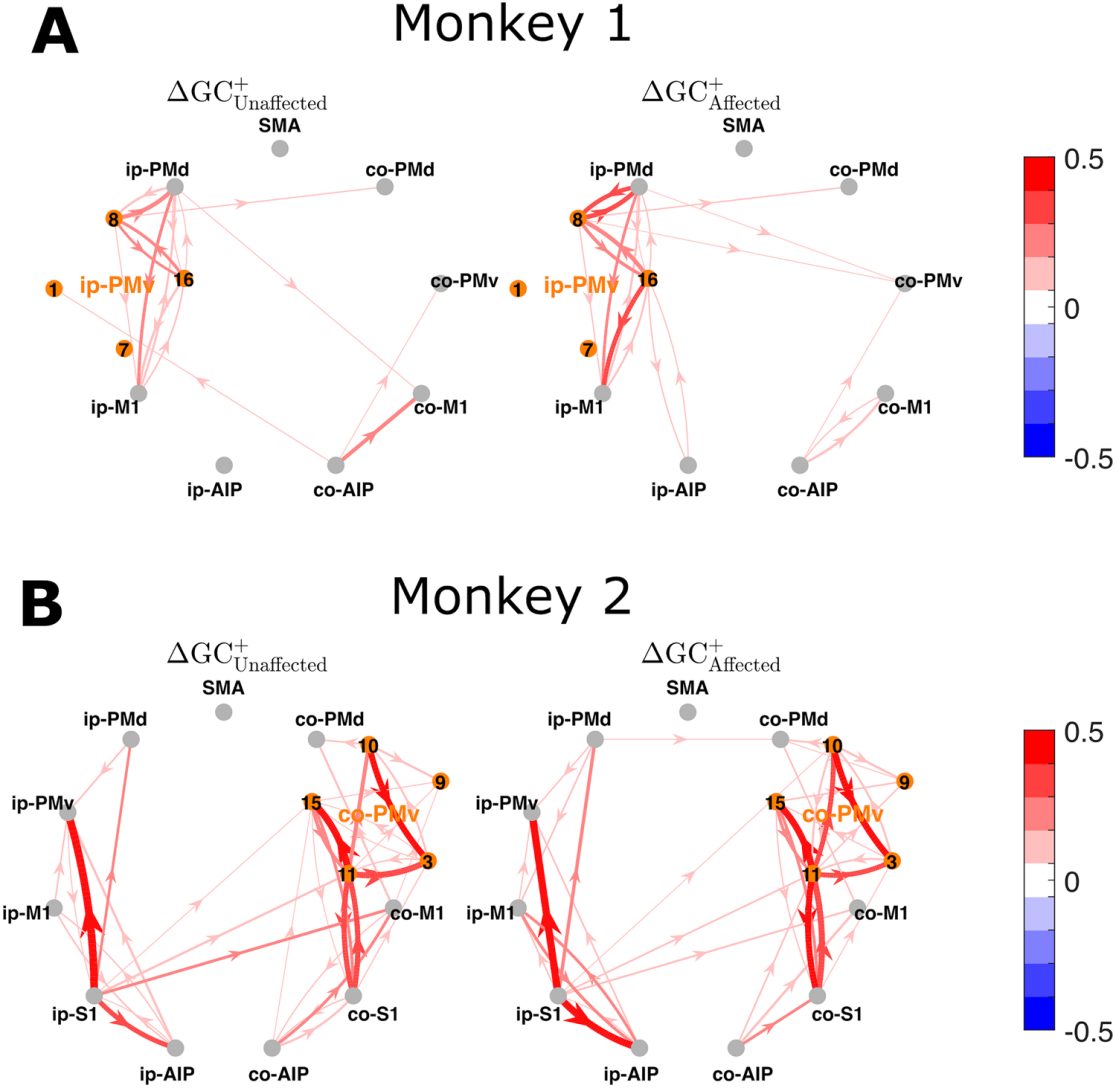
Directed graphs displaying the region-level total increases in GC, with every channel in (***A***) ip-PMv (Monkey 1) or (***B***) co-PMv (Monkey 2) treated as a single node. Only statistically significant edges are shown in each graph (permutation test, FDR = 0.1). For clarity, significant but small connectivity values are not shaded (color levels in white).

These observations are also intuitively confirmed by the directed graphs of region-level GC increases (Fig. 9), where each channel in ip-PMv (Monkey 1) or co-PMv (Monkey 2) is treated as a single node. In Monkey 1, Channels 8 and 16 clearly serve as “hubs” in these graphs, connecting PMd and M1 via PMv in the ipsilesional hemisphere. This is more pronounced for affected-hand than unaffected-hand use (Fig. 9*A*). In Monkey 2, Channel 11 functioned as a hub in the contralesional hemisphere, particularly within co-PMv and between co-PMv and co-S1 (Fig. 9*B*). Consequently, these target channels in their respective PMv areas, originally examined by Kato et al. (2020), were found to play a significant role in the pre–post dFC changes in both monkeys.

## Discussion

In the present study, fNIRS revealed differences in dFC between motor-related areas from preinfarction to postmotor function recovery. In Monkey 1, which had a small infarct, dFC changes were mainly observed between areas of the ipsilesional hemisphere, whereas in Monkey 2, which had a large infarct, dFC changes were observed in the ipsilesional hemisphere, between areas of the contralesional hemisphere and between areas in both hemispheres. These findings align with those of previous studies in patients with brain damage and animal models, in which increased activation and remodeling of neural structures in the motor cortex occurred in both the ipsilesional and contralesional hemispheres during functional recovery, and changes in the contralesional motor cortex were reported to be more pronounced when damage was more severe (Johansen-Berg et al., 2002; Hsu and Jones, 2006; Liu et al., 2008; Schaechter and Perdue, 2008; Bestmann et al., 2010; Rehme et al., 2011; Bradnam et al., 2012; Kantak et al., 2012; Touvykine et al., 2016; Dodd et al., 2017). In our previous study, which provided the fNIRS data for the present study, we reported enhanced activation in the ipsilesional PMv of the monkey with less extensive damage and in the contralesional PMv of the monkey with more extensive damage (Kato et al., 2020). It is unlikely that functional recovery after brain damage can be achieved solely through increased activity in one area. The present results are important because they demonstrate network changes involving multiple motor-related cortical areas. Moreover, our previous experiment involving pharmacological inactivation of PMv areas after recovery led to a reinduction of motor impairments (Kato et al., 2020). The present study, which included an analysis of degree centrality in the connectivity network, indicated that these areas served as a hub of information processing in motor-related cortical areas following recovery. These results suggest that functional connectivity analysis using fNIRS is effective for monitoring the functional brain changes underlying motor function recovery.

The results for Monkey 1 in the present study showed increased mutual dFC between the PMd and PMv areas and from the PMv area to M1 in the ipsilesional hemisphere after recovery, particularly when the affected hand was used. It is likely that some output fibers from M1, which pass through the posterior limb of the internal capsule in the ipsilesional hemisphere, remained intact in Monkey 1. The increased dFC from the premotor cortex may have played a role in maximizing the use of undamaged output pathways originating from M1. In Monkey 2, there was increased mutual dFC between the contralesional PMv, which exhibited heightened activity in our previous study (Kato et al., 2020), and S1 in the same hemisphere. An increase in dFC from S1 to PMv was also observed in the ipsilesional hemisphere.

These findings align with a previous study that reported enhanced neuronal projections between PMv and S1 in the same hemisphere following damage to the M1 hand region in a monkey (Dancause et al., 2005). Furthermore, functional brain imaging and physiological experiments in brain-damaged patients have indicated that the body representation in S1 is reorganized after recovery of motor function (Pineiro et al., 2001; Roiha et al., 2011). It is possible that changes in both the contralesional S1 and PMv occurred to provide somatosensory feedback to control the affected hand—the hand ipsilateral to the hemisphere—because somatosensory feedback is essential for generating appropriate motor output from the contralesional hemisphere (Williamson et al., 2024). The present results also indicate an increase in dFC in the AIP, especially when using the affected hand. Given the AIP’s important role in visuomotor transformations during grasping movements (Davare et al., 2011), reorganization of the functional relationship between this area and other motor-related areas may also have occurred during motor recovery. Although it is difficult to infer the motor output pathways from the motor cortex for Monkey 2 based on the results of the present study, we speculate that motor output from PMv via the subcortical motor nuclei may play a central role, because our previous study showed increased neuronal projections from PMv to the subcortical motor nuclei during motor recovery after M1 damage (Yamamoto et al., 2019).

In addition, dFC decreased in several areas after recovery. In both monkeys, one of the most notable findings was a reduction in dFC between the premotor areas and the SMA. Although the neurological significance of this finding is currently unclear, we speculate that the decrease may contribute to the inhibition of mirror movements—which are involuntary, synchronous movements of the unaffected hand that occur when the affected hand moves voluntarily (Cox et al., 2012)—because the SMA is involved in bimanual movements and mirror movements (Brinkman, 1981, 1984; Welniarz et al., 2019). Given that mirror movements are believed to stem from abnormal connections in neural pathways such as the corpus callosum and the corticospinal tract (Kuo et al., 2018; Liu et al., 2021), analyzing dFC in models in which mirror movements occur may offer valuable insights into the mechanisms underlying their emergence. Moreover, in Monkey 2, there was a decrease in dFC between all corresponding cortical areas examined across both hemispheres after recovery. Numerous studies have indicated that interhemispheric inhibition is altered during motor recovery following brain damage (Boddington and Reynolds, 2017; Gerges et al., 2022; Mirdamadi et al., 2023). Although much attention has focused on interhemispheric inhibition between M1 in both hemispheres, the present results highlight the contribution of interhemispheric inhibition in other sensorimotor-related areas.

Several previous studies have reported changes fNIRS in functional connectivity in stroke patients (Wang et al., 2010; Park et al., 2011; Grefkes and Fink, 2014; Arun et al., 2020; Cassidy et al., 2022; Zou et al., 2023; Ismail et al., 2024). Most of these investigations have analyzed rs-FC using rsfMRI or fNIRS. Although a direct comparison with the present study, which focuses on task-dependent functional connectivity changes, is not straightforward, some commonalities can be observed. For instance, studies comparing rs-FC between stroke patients and healthy controls using fNIRS have reported decreased interhemispheric functional connectivity in stroke patients (Zou et al., 2023), a finding consistent with the results for Monkey 2 in this study. Furthermore, reports that functional connectivity among cortical regions related to somatosensory and motor processing—including the PM, M1, and S1—increases during the process of motor recovery also align with the present findings (Wang et al., 2010; Arun et al., 2020; Ismail et al., 2024). An important advantage of previous studies analyzing rs-FC is that they do not require task performance, thus enabling the evaluation of longitudinal changes during the course of motor recovery. Future studies using simpler tasks that can be performed before full recovery of motor function may elucidate longitudinal changes in task-dependent functional connectivity changes during functional recovery.

There are several limitations of the present study that need to be addressed. First, the ROIs were determined based on the location of optodes on the MRI; however, each ROI may not correspond exactly to a single cortical area. For example, activity in the ROI designated as AIP is considered to include activity in the adjacent Area 5. Future studies using diffuse optical tomography (DOT), which provides a three-dimensional image reconstruction of brain activity from fNIRS data mapped onto an anatomical image (Bluestone et al., 2001; Hayashi et al., 2022), will allow for a more precise analysis of dFC changes between specific areas or subareas. Furthermore, the present analysis could not analyze the medially located SMAs in the ipsilesional and contralesional hemispheres due to a lack of fNIRS signal sensitivity, which hindered the interpretation of the dFC changes underpinning motor recovery. We believe that DOT with high spatial resolution will enable separate analyses of the ipsi- and contralesional SMAs. Second, we focused only on changes in oxygenated hemoglobin, although fNIRS can simultaneously measure changes in the concentrations of both oxygenated and deoxygenated hemoglobin. The use of multivariate GC to analyze changes in deoxygenated hemoglobin, total hemoglobin, and oxygenated hemoglobin would provide a more comprehensive picture of dFC changes that occur during the functional recovery process. Finally, as shown in Figure 6, we obtained the time-integrated increases and decreases in GC for shorter (4 s) time windows, using the superior temporal resolution of fNIRS compared with fMRI. This analysis revealed characteristic dFC changes in each time window; for example, the increase in dFC involving the contralesional PMv preceded that involving the ipsilesional PMv while using the affected hand in Monkey 2. These results have implications regarding changes in the flow of information from motor command generation to motor output. However, the physiological interpretation is challenging because there are delays between changes in neural activity and hemodynamics measured by fNIRS, and the physiological mechanisms underlying these delays are not fully understood (Buxton et al., 2004; Ma et al., 2016). Future studies using electrophysiological neural activity measurements in model animals combined with fNIRS should clarify the temporal relationship between neural activity and changes in hemodynamics.
